# Education Research: Trends in Faculty Tenure Status and Diversity in Academic Neurology Departments in the United States

**DOI:** 10.1212/NE9.0000000000200272

**Published:** 2025-11-14

**Authors:** Dheeman Futela, Keervani Kandala, Huanwen Chen, Marco Colasurdo, Dheeraj Gandhi, Ajay Malhotra

**Affiliations:** 1 Yale School of Medicine, New Haven, CT;; 2 Yale University, New Haven, CT;; 3 National Institute of Neurological Disorders and Stroke, Bethesda, MD;; 4 OHSU, Portland, OR;; 5 University of Maryland Medical Center, Baltimore, MD;; 6 Yale University School of Medicine, New Haven, CT.

## Abstract

**Background and Objectives:**

Tenure has long been fundamental to academic medicine, supporting faculty advancement and academic freedom. However, tenure-track positions in US medical schools have declined sharply, raising concerns for academic departments including neurology, a field reliant on a stable workforce to drive research, education, and patient care. This study examines trends in tenure status among academic neurology faculty from 2000 to 2023, focusing on gender and racial and ethnic diversity.

**Methods:**

Using the AAMC Faculty Roster through the FAMOUS portal, full-time academic neurology faculty tenure status was classified by gender and race and ethnicity. Underrepresented in medicine (URiM) faculty were defined as American Indian or Alaska Native, Black or African American, Hispanic, or Native Hawaiian, whereas non–underrepresented in medicine (non-URiM) faculty included all other racial and ethnic groups such as Asian, Other Race, Multiple Race, Non-Hispanic, White, and Unknown. Statistical analyses included linear regression for trends and multiple logistic regression to analyze the effects of gender and race and ethnicity on odds of tenure-line status over time.

**Results:**

From 2000 to 2023, the number of full-time academic neurology faculty more than doubled (3,149–7,129), and the proportion of non-URiM men declined (69%–51%), whereas the proportion of non-URiM women increased (26%–40%) and URiM faculty increased (5%–9%) (*p* < 0.001). Adjusting for gender and race and ethnicity, the odds of tenure-line status decreased over time (odds ratio 0.96 per year, *p* < 0.001). The odds of tenure-line status for all subgroups were lower than for White men throughout the study period. At the current trajectory, this gap will close with time for URiM women (until 2040), Asian women (until 2049), and White women (until 2053), but not for Asian men and URiM men. In 2023, compared with Internal Medicine and Surgery departments, Neurology had greater representation of women, lower URiM representation, and a higher proportion of tenure-line faculty.

**Discussion:**

Despite faculty expansion, the proportion of tenure-line positions in academic neurology declined, with persistent underrepresentation of women and URiM faculty, possibly reflecting persistent structural barriers in academic advancement that need to be addressed.

## Introduction

Tenure has traditionally played a critical role in academic medicine, shaping faculty advancement and ensuring academic freedom.^1^ However, over the past few decades, US medical schools have seen a decline in tenure-track positions. Specifically, the percentage of all clinical faculty on tenure-eligible tracks decreased from 59% in 1982 to just 18% in 2022.^2^ This trend is driven by various factors, including evolving health care delivery models, changes in reimbursement policies, and institutional budget constraints. As a result, academic institutions face immense pressure to prioritize clinical revenue generation over educational and research investments, leading to a significant expansion of non–tenure-track faculty positions. These changes are not uniform across all medical specialties. Neurology relies heavily on a stable academic workforce to drive research, train future physicians, and improve care for complex neurologic conditions.^3^ However, the tenure trends in academic neurology faculty, particularly regarding gender and race and ethnicity representation, remain underexplored. Disparities in tenure could influence career progression, leadership representation, and mentorship opportunities for future generations. These disparities do not occur in isolation but are influenced by structural racism, systemic inequities, and historical barriers within academic medicine, which have long shaped opportunities for faculty advancement and access to tenure, raising important questions of racial equity and inclusion. In this study, we aim to examine broader trends in tenure-line positions within academic neurology from 2000 to 2023 and assess disparities in representation by gender and race and ethnicity, recognizing these as interrelated but distinct aspects of academic workforce development.

## Methods

### Data Source

This study was a secondary analysis of the AAMC Faculty Roster using the online FAMOUS (Faculty Administrative Management Online User System) portal, a comprehensive national database of LCME-accredited US medical schools.^4^ The number and proportion of faculty by gender, race and ethnicity, and tenure status for neurology were collected from 2000 to 2023.

### Study Population

The faculty roster categorizes tenure status into 5 groups: tenured, on a tenure track, not on a tenure track, tenure not available, and missing tenure information. Tenure not available refers to faculty at medical schools that do not offer tenure, and missing refers to cases with omitted tenure status. In this study, we combined faculty who are tenured or on a tenure track as “tenure-line” faculty. Tenure status was compared across faculty according to (i) gender: AAMC reports “gender” to include self-reported binary gender information and (ii) race and ethnicity: the following mutually exclusive racial and ethnic groups were included in the analysis—Hispanic or Latino (of any race), Asian, Black or African American, White, American Indian or Alaska Native, Native Hawaiian, Other Race, Multiple Race, Non-Hispanic, and Unknown. Underrepresented in medicine (URiM) faculty were defined as American Indian or Alaska Native, Black or African American, Hispanic, or Native Hawaiian while non–underrepresented in medicine (non-URiM) faculty included all other racial and ethnic groups such as Asian, Other Race, Multiple Race, Non-Hispanic, White, and Unknown.

### Statistical Analysis

Percentage representation of subgroups by gender and race and ethnicity was plotted from 2000 to 2023, and linear regression models were fit to assess statistical significance of the shift in representation over time. Logistic regression analysis was performed to analyze odds of tenure-line status over time and to assess the effect of subgroups by gender and race and ethnicity (considering White men as reference). Interaction terms (subgroup * year) were included to assess whether the trend in odds of tenure-line status varied significantly for different subgroups. For subgroups with significant interactions, predicted probability of tenure-line was projected from 2023 onward and the time to parity with White men was calculated. Finally, tenure status and diversity (gender and racial/ethnic) in Neurology was compared with Internal Medicine and Surgery departments using the χ^2^ test for significance. All statistical analyses and graphing were performed with *Python v3.1.2*.

### Data Availability

The AAMC Faculty Roster data are publicly available (at a cost to AAMC members and nonmembers).

### Standard Protocol Approvals, Registrations, and Participant Consents

This study was deemed exempt from human subject research guidelines by the IRB because it involved only deidentified data.

## Results

**Figure 1:**
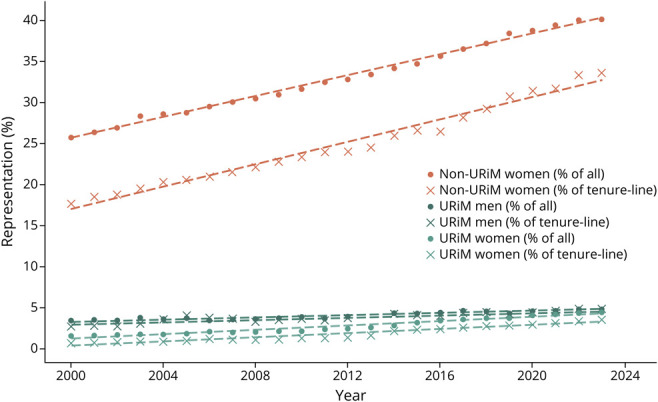
Representation Trends Among Neurology Faculty Trends in representation (%) of women and URiM groups among all faculty (dots) and among tenure-line faculty (crosses). Dashed line shows the linear regression model for each group. URiM faculty were defined as American Indian or Alaska Native, Black or African American, Hispanic, or Native Hawaiian, whereas non-URiM faculty included all other racial and ethnic groups such as Asian, Other Race, Multiple Race, Non-Hispanic, White, and Unknown. URiM = underrepresented in medicine.

From 2000 to 2023, the number of full-time academic neurology faculty in the United States more than doubled from 3,149 to 7,129. Table 1 summarizes the demographics of full-time academic neurology faculty in 2023. The proportion of non-URiM men decreased significantly during the study period but remained a majority throughout (69%–51%, −0.8% annually, *p* < 0.001). The proportion of non-URiM women increased (26%–40%, 0.6% annually, *p* < 0.001), and the proportions of URiM faculty increased (5%–9%, 0.2% annually, *p* < 0.001) (Figure 1). Overall, the proportion of White faculty decreased (80%–60%, −0.84% annually, *p* < 0.001), whereas the representation of Asian (11%–24%, 0.56% annually, *p* < 0.001), Black (1.4%–2.6%, 0.06% annually, *p* < 0.001), and Hispanic (3.5%–6.4%, 0.13% annually, *p* < 0.001) faculty increased (Table 2).

**Table 1:** Number of Full-Time Academic Neurology Faculty in 2023 According to Gender, Race and Ethnicity, and Tenure-Line (Including Tenured and On-Tenure-Track) Status

Category	Total (n = 7,129)	Tenure-line status (n = 1,903)
Gender		
Men	3,954 (55.5%)	1,197 (62.9%)
Women	3,175 (44.5%)	706 (37.1%)
Race and ethnicity		
Asian	1,683 (23.6%)	381 (20%)
American Indian, Alaska Native, or Native Hawaiian	17 (0.2%)	3 (0.2%)
Black or African American	186 (2.6%)	46 (2.4%)
Hispanic (any race)	457 (6.4%)	110 (5.8%)
White	4,286 (60.1%)	1,266 (66.5%)
Others (Other Race, Multiple Race, Non-Hispanic, and Unknown)	500 (7%)	97 (5.1%)

**Table 2:** Trends in Representation (%) Among Full-Time Academic Neurology Faculty According to Gender and Race and Ethnicity

Category	Representation (%) in academic neurology faculty		Linear regression model: change in representation (%) over time		
	2000 (n = 3,149)	2023 (n = 7,129)	Slope (% pts per year)	95% confidence interval	*p* Value
Asian men	7.7% (243/3,149)	13% (926/7,129)	0.22	0.19 to 0.25	<0.001
Asian women	2.9% (92/3,149)	10.6% (757/7,129)	0.35	0.34 to 0.37	<0.001
URiM^a^ men	3.4% (108/3,149)	4.8% (345/7,129)	0.07	0.06 to 0.08	<0.001
URiM^a^ women	1.5% (47/3,149)	4.4% (315/7,129)	0.13	0.12 to 0.15	<0.001
White men	58.8% (1853/3,149)	34.3% (2,442/7,129)	−1.06	−1.08 to −1.03	<0.001
White women	21.4% (673/3,149)	25.9% (1844/7,129)	0.22	0.2 to 0.24	<0.001

a URiM (underrepresented in medicine) includes Hispanic, Black/African American, American Indian/Alaska Native, and Native Hawaiian populations.

The total number of non–tenure-line faculty increased 3-fold (1,573–5,208), and the number of tenure-line (tenured or on-tenure-track) faculty increased by roughly 50% (1,281–1903). The number of tenure-line faculty decreased among White men but increased across all other subgroups. This resulted in significant shift in representation of all subgroups among tenure-line faculty (*p* < 0.001 for all) (Table 3). The representation of White men decreased from 70% to 43% (−1.1% annually), largely replaced by White women (15%–23%, 0.3% annually), Asian women (1.6%–7.8%, 0.29% annually), Asian men (7%–12%, 0.22% annually), URiM women (0.6%–3.5%, 0.13% annually), and URiM men (2.7%–4.8%, 0.08% annually) (Figure 1).

**Table 3:** Trends in Representation (%) Among Tenure-Line (Including Tenured and On-Tenure-Track) Neurology Faculty According to Gender and Race and Ethnicity

Category	Representation (%) in tenure-line faculty		Linear regression model: change in representation (%) over time		
	2000 (n = 3,149)	2023 (n = 7,129)	Slope (% pts per year)	95% confidence interval	*p* Value
Asian men	7% (90/1,281)	12.2% (233/1903)	0.22	0.2 to 0.25	<0.001
Asian women	1.6% (20/1,281)	7.8% (148/1903)	0.29	0.27 to 0.31	<0.001
URiM^a^ men	2.7% (35/1,281)	4.8% (92/1903)	0.08	0.06 to 0.1	<0.001
URiM women	0.6% (8/1,281)	3.5% (67/1903)	0.13	0.11 to 0.14	<0.001
White men	70.1% (898/1,281)	43% (819/1903)	−1.16	−1.21 to −1.1	<0.001
White women	15.4% (197/1,281)	23.5% (447/1903)	0.34	0.3 to 0.38	<0.001

a URiM (underrepresented in medicine) includes Hispanic, Black/African American, American Indian/Alaska Native, and Native Hawaiian populations.

**Figure 2:**
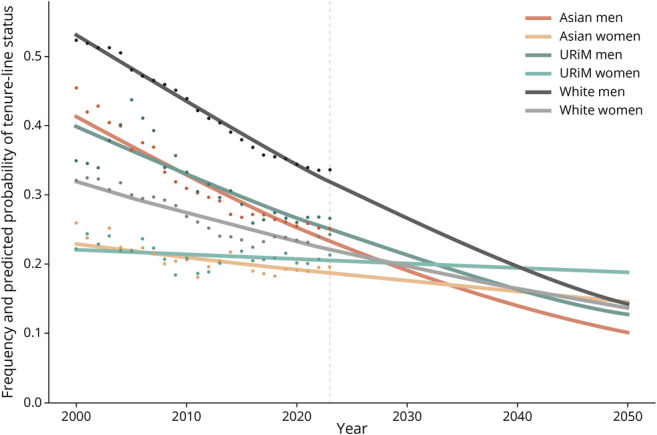
Probability of Tenure-Line Status for Neurology Faculty by Gender and Race and Ethnicity Trend lines after the year 2023 show projected probability using the logistic regression model. Dots show the observed value of tenure-line proportion between 2000 and 2023. URiM faculty were defined as American Indian or Alaska Native, Black or African American, Hispanic, or Native Hawaiian, whereas non-URiM faculty included all other racial and ethnic groups such as Asian, Other Race, Multiple Race, Non-Hispanic, White, and Unknown. URiM = underrepresented in medicine.

The proportion of tenure-line faculty (of all faculty) decreased from 41% to 27% overall and decreased within each subgroup, except URiM women (increased from 17% to 21%). The odds ratios (ORs) of tenure-line status according to gender, race and ethnicity, and years are summarized in Table 4. Adjusting for gender and race and ethnicity, the odds of tenure-line status decreased over time (OR 0.962 per year [95% CI 0.960–0.964], *p* < 0.001). In the year 2023, compared with White men as reference, the odds of tenure-line status were significantly lower for all other groups by race and ethnicity and gender (*p* < 0.001), in the following order: URiM men (OR 0.71 [95% CI 0.64–0.79]), Asian men (0.65 [0.60–0.70]), White women (0.61 [0.57–0.64]), URiM women (0.55 [0.48–0.63]), and Asian women (0.49 [0.45–0.54]) (Table 4). Between 2000 and 2023, the odds ratio of tenure-line status compared with White men narrowed significantly for White, Asian, and URiM women (interaction *p* < 0.001 for all 3) but not for Asian men (interaction *p* = 0.57) and URiM men (interaction *p* = 0.09). Therefore, at the current trajectory, the probability of tenure-line status will reach parity with White men in the year 2040 for URiM women, in 2049 for Asian women, and in 2053 for White women (Figure 2).

**Table 4:** Odds of Tenure-Line Status According to Subgroups by Gender and Race and Ethnicity and Over Time

	OR [95% confidence interval]	*p* Value
Years	0.962 [0.96–0.965]	<0.0001
Subgroup (reference year 2023)		
White men	1 (reference)	
White women	0.61 [0.57–0.64]	<0.0001
Asian men	0.65 [0.60–0.70]	<0.0001
Asian women	0.49 [0.45–0.54]	<0.0001
URiM^a^ men	0.71 [0.64–0.79]	<0.0001
URiM women	0.55 [0.48–0.63]	<0.0001
Interaction		
White women^a^ years	1.017 [1.012–1.021]	<0.0001
Asian men^a^ years	1.002 [0.996–1.008]	0.573
Asian women^a^ years	1.028 [1.019–1.037]	<0.0001
URiM men^a^ years	1.008 [0.999–1.018]	0.092
URiM women^a^ years	1.035 [1.021–1.049]	<0.0001

Odds ratios (ORs) are derived from the logistic regression model centered on the year 2023. *p* values < 0.05 show significant main effect or interaction effect on the odds of tenure-line status compared with the reference.

a URiM (underrepresented in medicine) includes Hispanic, Black/African American, American Indian/Alaska Native, and Native Hawaiian populations.

In the year 2023, the representation of women in Neurology (44.5%) was greater than in Internal Medicine (42.9%, *p* = 0.01) and Surgery (29.1%, *p* < 0.001) departments, whereas the representation of URiM faculty in Neurology (9.3%) was lower than in Internal Medicine (10.0%, *p* = 0.04) and Surgery (10.2%, *p* = 0.03) departments. The proportion of tenure-line faculty in Neurology (26.7%) was greater than in Internal Medicine (20.2%, *p* < 0.001) and Surgery (21.6%, *p* < 0.001). Compared with Neurology, only 4 clinical specialties had a greater proportion of tenure-line faculty—Public Health/Preventative Medicine (32.5%), Ophthalmology (29.3%), Pathology (28.5%), and Otolaryngology (28.5%).

## Discussion

Between 2000 and 2023, the number of neurology faculties across US medical schools more than doubled. This study highlights 2 interrelated but distinct issues: the decline in tenure-line positions across academic neurology and the persistent disparities in tenure representation by gender and race and ethnicity. While the overall size of the neurology faculty workforce has expanded, the proportion of tenure-line positions has steadily decreased, raising important questions about the evolving structure of academic medicine.

Consistent with national trends, our results show an increasing shift toward non–tenure-line positions in neurology, reflecting institutional pressures to prioritize clinical revenue over research and education. These findings align with previous reports documenting the financial and structural drivers of tenure decline in academic medicine.^5,6^ The increasing prioritization of clinical service over research and education has resulted in a workforce model that heavily relies on non–tenure-line faculty. Mergers and acquisitions of physician practices and academic hospitals into health enterprises create challenges, increasing dependence of medical schools on affiliated clinical enterprises for financial support.^7^ There has been an increase in the percentage of medical schools from 29% in 2002 to 42% in 2022 that do not have any financial guarantees for tenure.^8^ Medical schools that do have financial guarantees of tenure for clinical faculty have shifted away from base salary to fixed-dollar or standard-referenced amounts.^8^ The growing ranks of clinicians with faculty appointments who have fewer teaching and research responsibilities, as well as medical schools' financial models and higher salary structure, may be contributing factors leading to decline in tenure.^8^ Newer medical schools, especially those not associated with a university, are less likely to adopt the tenure structure of academia.^8^ These trends raise concerns, given that tenure-line faculty have historically been essential in advancing research and mentoring future generations of physician-scientists.^9,10^ However, it is important to recognize that tenure is no longer the only pathway to academic success.

Many institutions have created alternative promotion and career development structures for non–tenure-line faculty, including clinician-educator tracks, research contracts, and endowed positions, which provide meaningful opportunities for professional advancement. For some clinician-educators, the reduced emphasis on tenure is viewed positively, as it creates greater equity between faculty whose primary contributions are in teaching or clinical care and those in research. Therefore, the decline of tenure has both risks and potential benefits: it may threaten long-term investment in research and academic freedom, but it also opens the door for broader recognition of diverse faculty roles.

Despite the overall increase in gender and racial and ethnic diversity among neurology faculty, disparities in tenure-line representation persist. Women and faculty URiM remain disproportionately excluded from tenure tracks, limiting their access to promotion, leadership roles, and mentorship opportunities.

These disparities are not simply the result of individual career choices but are shaped by structural racism, systemic inequities, and historical barriers that have long influenced advancement in academic medicine.^11,12^ For example, women and URiM faculty faced unequal access to mentorship and sponsorship opportunities (such as the “minority tax,” where URiM faculty are disproportionately asked to serve on committees and diversity efforts at the expense of scholarly productivity). Differential allocation of research funding has been well documented, with studies showing lower NIH funding rates for Black investigators compared with White investigators, even after accounting for previous productivity and institution.^13^ Historical exclusion of racial and ethnic minority groups from medical training and leadership positions continues to influence today's academic hierarchies.^14,15^ Gender inequities persist in promotion and tenure, with women less likely to advance to full professor or leadership roles, even when controlling for qualifications and productivity.^16^ Together, these systemic factors highlight that inequities in tenure representation reflect institutional and structural barriers rather than individual preferences alone. Addressing these inequities requires more than recruitment and retention policies; it necessitates deliberate, sustained efforts to dismantle structural barriers; confront systemic racism; and embed principles of racial and gender equity into promotion, mentorship, funding, and leadership structures across academic neurology.

To address systemic inequities in academic neurology, institutions may use strategies such as tenure-clock flexibility, implicit bias training for promotion and tenure committees, and institutional policies that support career development for women and URiM faculty that could help mitigate disparities. In addition, efforts to redefine tenure policies, including post-tenure review processes and financial restructuring, may improve career stability and research engagement for faculty across all demographic groups.^17-19^Broader institutional reforms are also critical: transparent and consistently applied promotion criteria across all tracks; formal mentorship and sponsorship networks designed to support women, URiM faculty, and junior scholars; equity-focused approaches to allocating research funding and protected time; and investment in leadership development programs that prepare diverse faculty for senior roles. In parallel, institutions should recognize that nontenure tracks, such as clinician-educator pathways, long-term research contracts, and endowed positions, can provide meaningful stability, career satisfaction, and opportunities for advancement when they are structured with clear criteria and equitable access to resources. Redefining the role of tenure within this broader ecosystem, by embedding principles of accountability, inclusivity, and financial sustainability, may help balance institutional priorities with the need to foster a diverse and thriving academic workforce.

Our study has several limitations. First, the AAMC Faculty Roster does not include faculty from osteopathic medical schools, potentially limiting generalizability. Second, tenure definitions vary across institutions, and our classification of tenured/on-tenure-track faculty may not capture nuanced institutional differences. In addition, faculty race and ethnicity data are self-reported and may not fully reflect the diversity of academic neurology. Furthermore, in the AAMC Faculty Roster through FAMOUS, gender was reported only in binary categories, which limits our ability to capture the experiences of nonbinary faculty and may underestimate disparities in tenure representation. Finally, the lack of data for part-time faculty prevented analysis of their tenure status and potential disparities.

In summary, our findings underscore the dual challenges facing academic neurology: the overall decline in tenure-line positions and the persistent inequities in representation among women and URiM faculty. By acknowledging both risks and potential benefits of tenure decline and by addressing the systemic barriers that underlie disparities, academic neurology can move toward a more equitable and sustainable future.

## Glossary

OR: odds ratio
URiM: underrepresented in medicine
